# Effects of Microplastics and Organic Fertilizer Regulation on Soil Dissolved Organic Matter Evolution

**DOI:** 10.3390/toxics12100695

**Published:** 2024-09-26

**Authors:** Cheng Li, Chunhai Wang, Le Liu

**Affiliations:** 1 National and Local Joint Engineering Research Center of Biomass Resource Utilization, College of Environmental Science and Engineering, Nankai University, Tianjin 300350, China; felixfelicis@163.com; 2 Tianjin High-Quality Agricultural Products Development Demonstration Center, Tianjin 301508, China; wangchunhai1968@163.com

**Keywords:** microplastics, organic fertilizer, DOM, humic acid

## Abstract

Microplastics are pollutants of global concern nowadays. However, the effects of microplastics addition to soil as a carbon source and the combined effects of microplastics and organic fertilizer on soil-dissolved organic matter (DOM) evolution are still unclear. This study focused on the evolution of DOM in soil with the addition of microplastics and investigated the variations in the content and composition of DOM in unfertilized and fertilized soil with different particle sizes of microplastics. It was observed that the TOC concentration of the soil DOM in the treatment with organic fertilizer and microplastics increased more (129.97–161.43 mg kg^−1^) than that in the treatment with microplastics alone (117.17–131.87 mg kg^−1^) and was higher than that in the original soil (95.65 mg kg^−1^). According to the humic acid relative abundance in DOM after 40 days of incubation, the humic acid relative abundance in DOM of the soil samples with microplastics and organic fertilizers addition was found to be higher than that in those with microplastic addition alone, reaching more than 80% in a short time. In conclusion, the TOC concentration of the soil DOM increased with the addition of microplastics, and the increase was more pronounced when organic fertilizers and microplastics were added together. Moreover, the soil humification increased to a higher level in the short term with the combined addition of microplastics and organic fertilizers, which was maintained during the long-term incubation process.

## 1. Introduction

The general definition of microplastics is plastics with particle sizes of less than 5 mm, various shapes, stable chemical compositions. They are not easily degraded and can easily accumulate in the ecosystem and affect the food chain [1]. As a result, microplastics have received widespread attention as a new type of environmental pollutant. Microplastics can enter the soil through film mulching, irrigation, etc., and organic fertilizers produced by composting can also carry microplastics into the soil [2], making agriculture a heavily affected area in terms of microplastic soil pollution. Research has shown that in a 30-day soil incubation experiment, 28% of polyethylene microplastics significantly increased the accumulation of dissolved organic matter (DOM), promoting the release of dissolved organic carbon, dissolved organic phosphorus, and dissolved organic nitrogen into the soil, and significantly increasing soil nutrient concentrations until 14 to 30 days [3]. Therefore, the impact of microplastic particles on soil depends on their concentration and incubation time. Microplastics entering the soil environment may lead to changes in soil enzyme activity, microbial biomass, community composition and structure, thereby affecting soil fertility and soil environment. Thus, more research is needed to fully elucidate the impact of microplastics on soil ecosystems, especially agricultural ecosystems.

Dissolved organic matter (DOM) is a mixture of water-soluble organic compounds with different molecular weights and structures that pass through a 0.45 μm filter membrane. It is one of the most active organic components in soil and participates in various biogeochemical processes, especially the global carbon cycle [4]. The main sources of soil DOM are soil organic matter, plant root residues and microbial excretions, and anthropogenic sources from exogenous substances [5]. Exogenous substances such as microplastics can change soil DOM by releasing derived DOM into the soil, adsorbing natural soil DOM into pore structures, and affecting soil microorganisms and enzyme activity [6]. Studies have shown that the long-term application of organic fertilizers can change the pH and organic matter content in soil [7], as well as increase the soil fertility and the content of dissolved organic carbon [8]. While most current studies focus on the impact of fertilization on the quantity of DOM, research on the changes of DOM composition under the influence of fertilizers and microplastics is lacking. The three-dimensional excitation and emission matrix fluorescence (3DEEM) technique is convenient and fast in detecting the fluorescence of samples and provides spectra describing the composition of fluorescent groups related to molecules, which can be used to analyze the composition of DOM [9].

The decomposition and transformation of DOM in the soil environment are closely related to soil physicochemical properties, which not only affect the material cycling in soil but also the migration and transformation of soil pollutants. The impact of microplastics introduction to soil on the variations of DOM is still unclear, and there is a lack of research on the combined effects of microplastics and organic fertilizers on the regulation of soil DOM evolution. This study focused on the evolution of DOM in soil with the addition of microplastics, investigated the variations in the content and composition of DOM in unfertilized and fertilized soil with different particle sizes of microplastics, and explored whether soil DOM was affected by the synergistic impacts of microplastics and fertilizer.

## 2. Materials and Methods

### 2.1. Setting of Culture Conditions

The experiment used soil samples from the campus of Nankai University, Jinnan District, Tianjin City, China. The soil was of a brown color and possessed remarkable stickiness. After removing large stones and plant debris, the soil was sieved through a 2 mm sieve after air-drying and used for further experiments. The mass percentage of soil moisture content determined by the gravimetric method was 31.4%. The soil pH was determined by a pH meter (HQ2200, Loveland, CO, USA) at a soil–water ratio of 1:2.5. The elemental contents of carbon, hydrogen, and nitrogen in the soil samples and fertilizer were determined using an automatic elemental analyzer (EA3000, Pavia, LOM, Italy). The soil particle size was measured using the densitometer method. The soil organic matter was measured using the potassium dichromate oxidation method. The available nitrogen content of soil and the total nitrogen content of fertilizer were measured by Kjeldahl nitrogen determination. The available phosphorus content of soil and the total phosphorus content of fertilizer were measured by spectrophotometry. The available potassium content of soil was measured by flame spectrophotometry and the total potassium content of fertilizer was measured by atomic absorption spectrophotometry.

The basic characteristics of the soil and fertilizer used in the experiment are shown in Table 1. Polyethylene (PE) microplastics were purchased from Huachuang Plastics Co., Ltd. in Dongguan City, China. They were pre-treated on a sterile operation table for 30 min under UV disinfection to reduce microbial influence. Organic fertilizer was purchased from Dahuan Biotechnology Co., Ltd. in Tianjin City, China. After removing large stones, the fertilizer was sieved through a 2 mm sieve for use in further experiments.

A total of 200 g of 2 mm sieved dry soil was placed into a pot with a diameter of 10 cm and a height of 10.5 cm. The soil was incubated at room temperature to simulate the natural environment. The incubation experiment was divided into two major groups, A and B, with 8 treatments. The soil in group A was treated with microplastics, while the soil in group B was treated with microplastics and organic fertilizer. The application rate of organic fertilizer referred to the field application rate of 30 t ha^−1^ [10], and the amount of organic fertilizer added to each treatment was 1% (i.e., 2 g). The amount of PE microplastics added was 1% of the dry weight of the soil (i.e., 2 g). Treatment with 1000-mesh PE microplastics was named 1000PE, and treatment with 30-mesh microplastics was named 30PE. Among them, four treatments (A1000PE, A30PE, B1000PE, B30PE) were incubated for 40 days, and another four treatments were incubated for 100 days. The samples incubated for 40 dayere named A1000PE-40, A30PE-40, B1000PE-40, and B30PE-40, while the samples incubated for 100 days were named A1000PE-100, A30PE-100, B1000PE-100, and B30PE-100. The original soil was named S.

On day 0 of incubation, sterilized distilled water was added to each treatment to make the water content in the soil samples account for 70% of the water-holding capacity. Sterilized distilled water was added every three days during the incubation period to replenish the soil moisture, and the amount of water added was adjusted to match the weight of the samples on day 0. Three replicates were set for each treatment.

### 2.2. DOM Extraction and Soil Physicochemical Analysis

The soil samples after incubation were mixed with deionized water at a ratio of 1:5 (*W*/*V*) and shaken on a horizontal shaker at 200 rpm for 12 h. The samples were centrifuged at 8000 rpm for 5 min to separate the solid and liquid phases. The supernatant was filtered through a 0.45 μm micro-pore membrane filter, and the filtered samples were stored at 4 °C for subsequent testing. The water-soluble organic carbon content in the samples was measured using a total organic carbon analyzer (multi N/C3100, Jena, TH, Germany). The samples were analyzed for DOM composition using a three-dimensional fluorescence spectrometer (F-7000, Hitachi, Tokyo, Japan). The fluorescence analysis parameters were set with an emission wavelength range from 250 nm to 550 nm and an excitation wavelength range from 200 nm to 450 nm. The slit width for both emission and excitation wavelengths was set at 5 nm. The elemental contents of carbon, hydrogen, and nitrogen in the soil samples were also determined using an automatic elemental analyzer (EA3000, Pavia, LOM, Italy).

### 2.3. Extraction and Analytical Methods of Microplastics

Microplastic particles were extracted from the soil using the floatation method for the 30-mesh PE microplastics [11]. The treatments A30PE-40, B30PE-40, A30PE-100, and B30PE-100 were selected. The extracted microplastics were observed for surface morphology using a scanning electron microscope (SEM) (TESCAN MIRA LMS, Brno, JHM, Czech Republic). The samples were dried, mounted on a copper plate using conductive tape, sputter-coated with gold (10 nm) for 160 s, and then directly observed under the SEM. Multiple images were taken at 200× and 2000× magnifications to obtain representative images. The functional groups of the microplastics were tested using Fourier-transform infrared spectroscopy (FTIR) (Thermo Scientific Nicolet iS20, Waltham, MA, USA). Due to the larger particle size of the microplastics, analysis was performed directly in ATR mode.

### 2.4. Data Analysis

The data are shown as mean ± standard deviation and were recorded using Microsoft Excel 2019. Origin 2019b was used for graphing. Statistical analyses were performed using ISAS 9.4 (SAS institute Inc., Cary, NC, USA). The difference in soil TOC between different treatments was assessed using Tukey’s post hoc test at a significance level of 0.05.

## 3. Results

### 3.1. Evolution of Soil DOM Composition

**Figure 1:**
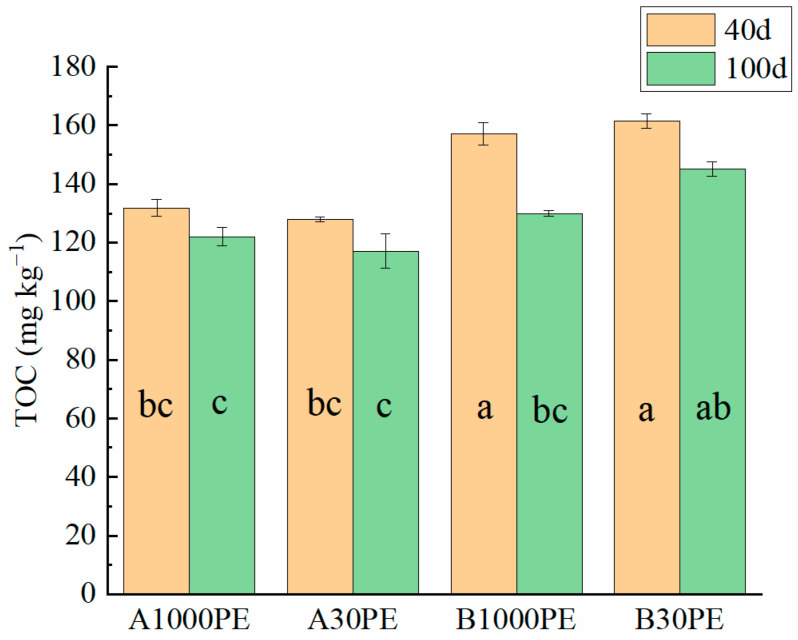
TOC concentration of DOM in different treatments. Same letters indicate no significant difference at *p* < 0.05 level.

The TOC concentrations of soil DOM in different treatments are shown in Figure 1. The TOC content of the original soil DOM was 95.65 mg kg^−1^. After the addition of microplastics and fertilizer, the TOC concentrations of soil DOM in all treatments increased. The increase in TOC concentration was more significant in the B group soil, which was treated with organic fertilizer and microplastics (129.97–161.43 mg kg^−1^), compared to the A group soil treated with only microplastics (117.17–131.87 mg kg^−1^). Comparing the soil samples incubated for 40 days and 100 days, the TOC concentrations of all groups decreased with time. However, after 100 days of incubation, the TOC concentration of soil DOM in the B group treated with organic fertilizer was still higher than that in the A group soil.

**Figure 2:**
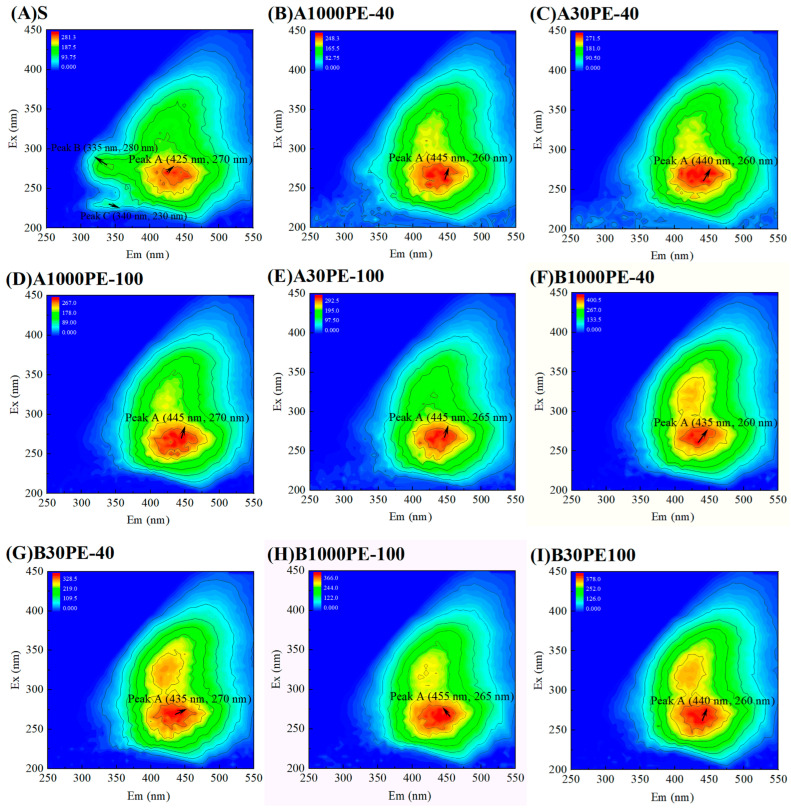
Three-dimensional excitation and emission matrix fluorescence spectra of DOM in treated soil and untreated soil.

The 3DEEM spectra of DOM in treated soil and untreated soil are shown in Figure 2. The spectra can clearly be used to identify three main fluorescence peaks: (1) Peak A: Ex/Em = (270/425 nm); (2) Peak B: Ex/Em = (280/335 nm); (3) Peak C: Ex/Em = (230/340 nm). Peak A had higher intensity and area, while Peaks B and C were connected to Peak A and had lower intensity. Compared to the original soil, the samples in both treatment groups showed similar characteristics, with Peaks B and C disappearing, and the peak shape of Peak A becoming clearer. The wavelengths of the peaks also shifted, indicating significant changes in the fluorescent component structure of the samples.

**Figure 3:**
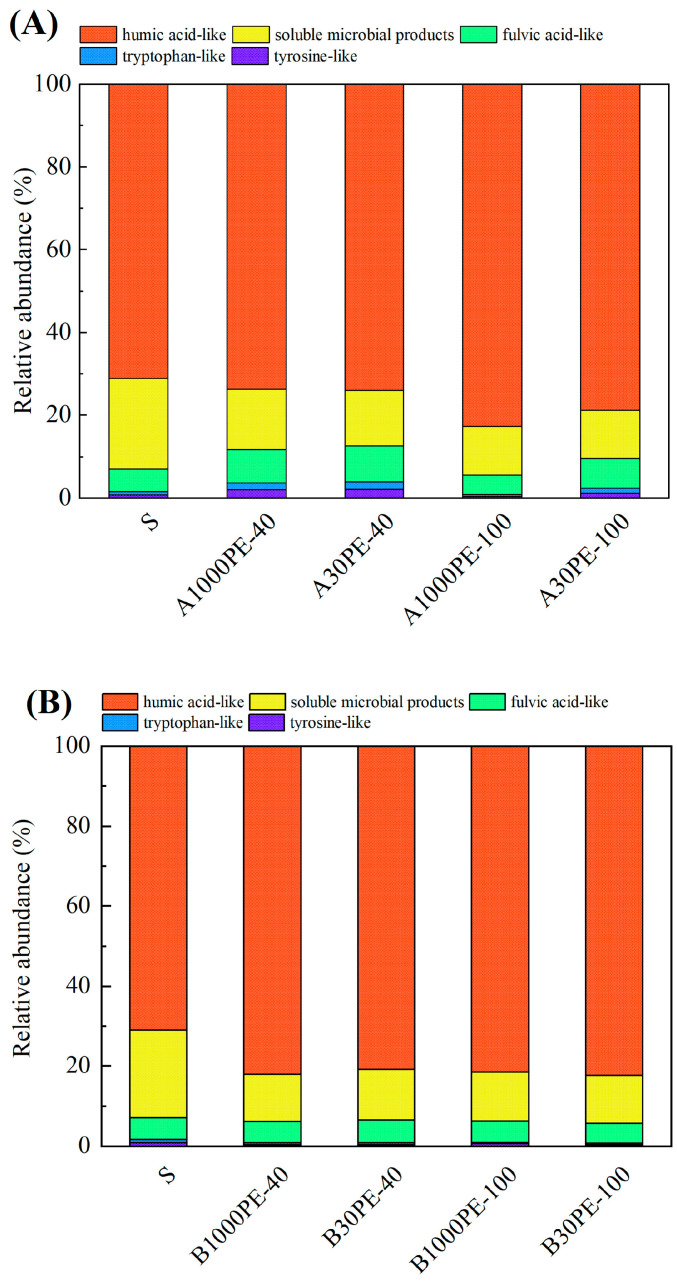
Relative abundance of three-dimensional fluorescent substances. (**A**): treatments with microplastics and (**B**): treatments with microplastics and organic fertilizer.

The 3D fluorescence spectra were divided into five regions based on the values of Ex and Em, which represented humic acid-like, soluble microbial products, fulvic acid-like, tryptophan-like and tyrosine-like substances [12]. The relative abundances of each region in each sample are shown in Figure 3.

The results showed that the relative abundance of humic acid-like substances in the original soil DOM was approximately 71.08%. In the treatment groups with added microplastics, the relative abundance of this component slightly increased, with the increase ranging from 2.58% to 11.55%. It indicates that there was a certain aggregation process of small-molecule substances in the samples, leading to the evolution of the entire component towards a more stable structure. In the soil samples taken at 40 days and 100 days, the relative abundance of humic acid-like substances in the soil DOM with added microplastics and organic fertilizer was generally higher than that in the soil DOM with only microplastics, and the relative abundance reached above 80% in all cases. Comparing A1000PE-40 (73.66%) and A1000PE-100 (82.63%), as well as A30PE-40 (73.90%) and A30PE-100 (78.82%), the relative abundance of humic acid-like substances in the soil DOM with added microplastics increased significantly with prolonged incubation time. Comparing B1000PE-40 (82.03%) and A1000PE-40 (73.66%), as well as B30PE-40 (80.91%) and A30PE-40 (73.90%), the relative abundance of humic acid-like substances in the soil DOM indicated that the addition of fertilizer could increase the relative abundance of humic acid-like substances in the early stage of incubation. Comparing B1000PE-40 (82.03%) and B1000PE-100 (81.46%), as well as B30PE-40 (80.91%) and B30PE-100 (82.35%), the relative abundance of humic acid-like substances in the soil DOM indicated that the addition of fertilizer could maintain a stable and higher level of relative abundance in the long-term incubation process.

**Figure 4:**
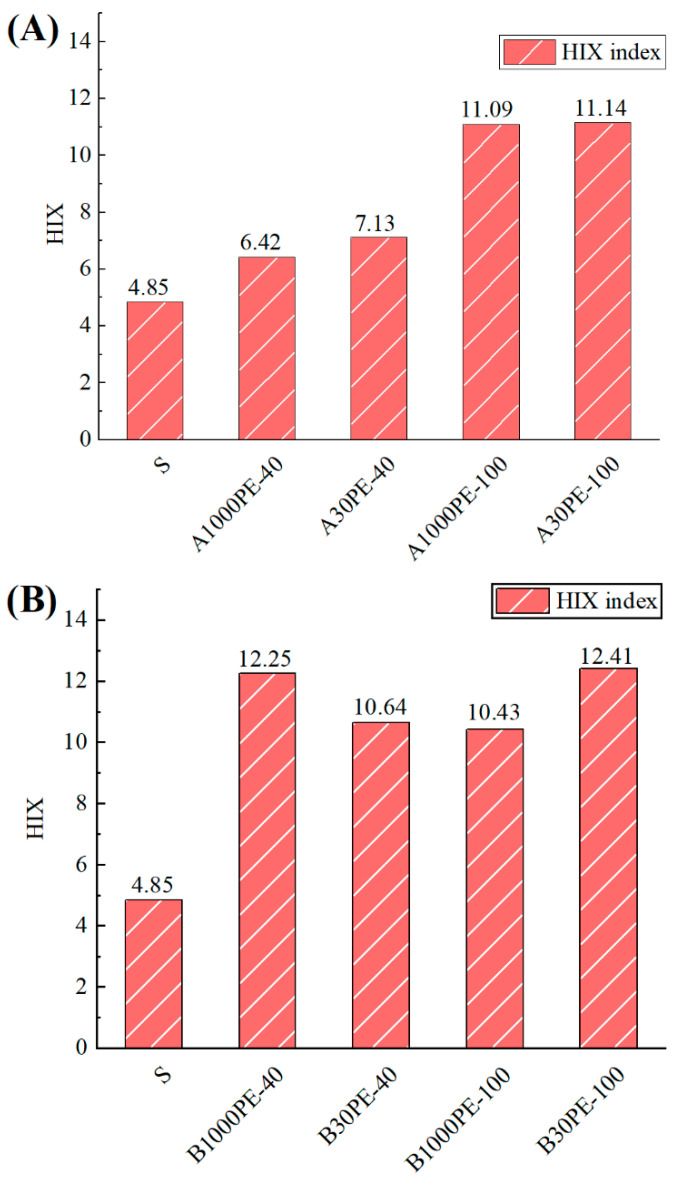
Humification index of dissolved organic matters in untreated soil and different treatments. (**A**): treatments with microplastics and (**B**): treatments with microplastics and organic fertilizer.

The humification index (HIX), defined as the fluorescence intensity in the 300–345 nm region divided by the sum of intensity in the 300–345 nm and 435–480 nm regions [13], is generally used to characterize the degree of humification in soil. As shown in Figure 4, the HIX of the A group increased significantly with prolonged incubation time. Comparing B1000PE-40 (12.25) and A1000PE-40 (6.42), as well as B30PE-40 (10.64) and A30PE-40 (7.13), the HIX indicated that the addition of fertilizer could increase the incubated degree of humification in the early stage of incubation. Comparing B1000PE-40 (12.25) and B1000PE-100 (10.43), as well as B30PE-40 (10.64) and B30PE-100 (12.41), the HIX indicated that the addition of fertilizer could maintain a consistently higher level of humification throughout the long-term incubation process.

### 3.2. Soil Elemental Composition

**Figure 5:**
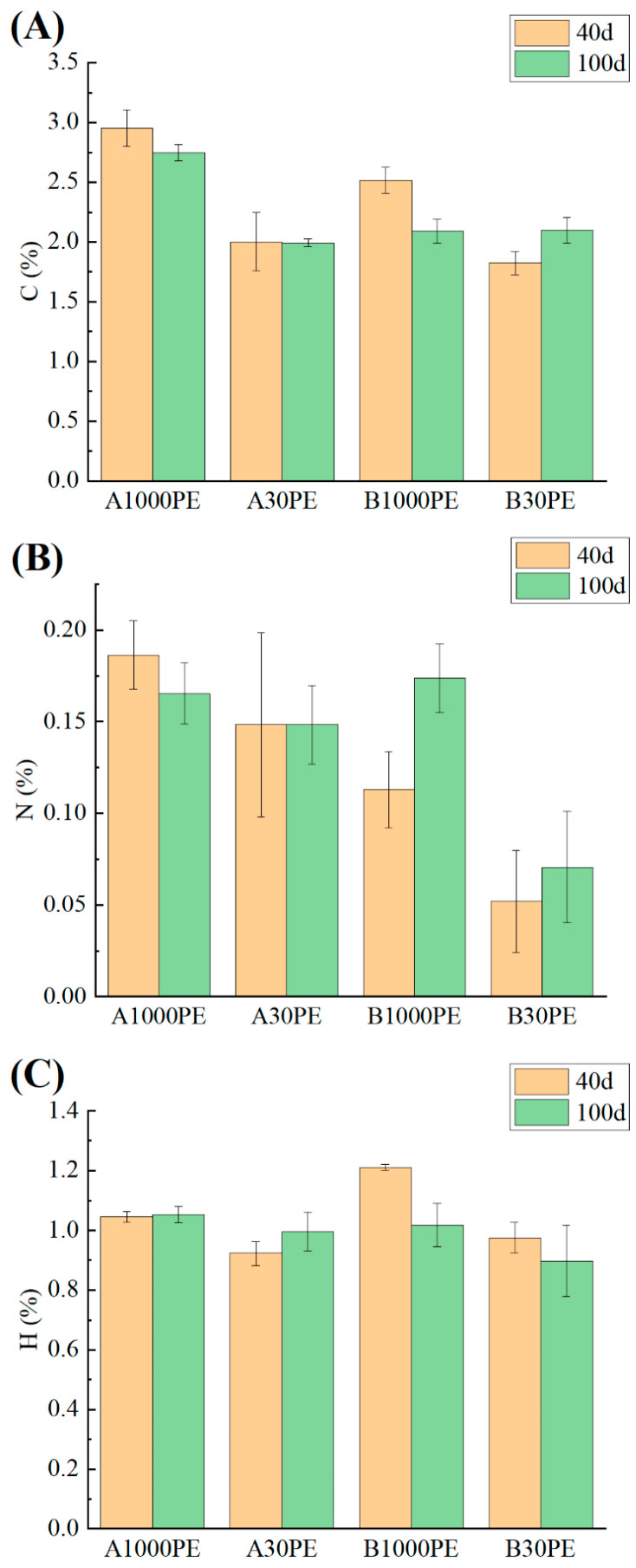
C (**A**), N (**B**), H (**C**) elemental content of different treatments.

The elemental contents of carbon, nitrogen, and hydrogen in each treatment are shown in Figure 5. The relative abundance of carbon in all treatment groups was higher than that in the original sample (1.790%), indicating that the addition of microplastics and fertilizer increased the relative carbon abundance in the soil. Except for the B30PE group, the carbon content in all other treatment groups decreased with prolonged incubation time, which may be related to the activity of soil microbial communities and carbon metabolism. The relative abundance of nitrogen in all treatment groups was lower than that in the original sample (0.254%). Under the same conditions, the relative nitrogen abundance in the treatment group with 30-mesh microplastics was lower than that in the treatment group with 1000-mesh microplastics. After 40 days of incubation, the decrease in the relative nitrogen content in Group B was greater than that in Group A compared to the original sample. After 100 days of incubation, the relative nitrogen abundance in Group A1000PE continued to decrease, while Group A30PE remained stable. The nitrogen content in Group B showed some recovery, and the increase in the relative nitrogen abundance in Group B1000PE was more pronounced than that in Group B30PE, indicating that the treatment group with larger particle size microplastics had a smaller change in relative nitrogen abundance. Except for the slightly lower carbon content in B1000PE-100 than in B30PE-100 under the same conditions, the relative abundance of carbon, hydrogen, and nitrogen in the treatment group with 30-mesh microplastics was lower than that in the treatment group with 1000-mesh microplastics.

### 3.3. Morphology and Composition Variations of Microplastics

#### 3.3.1. Morphology Variation

**Figure 6:**
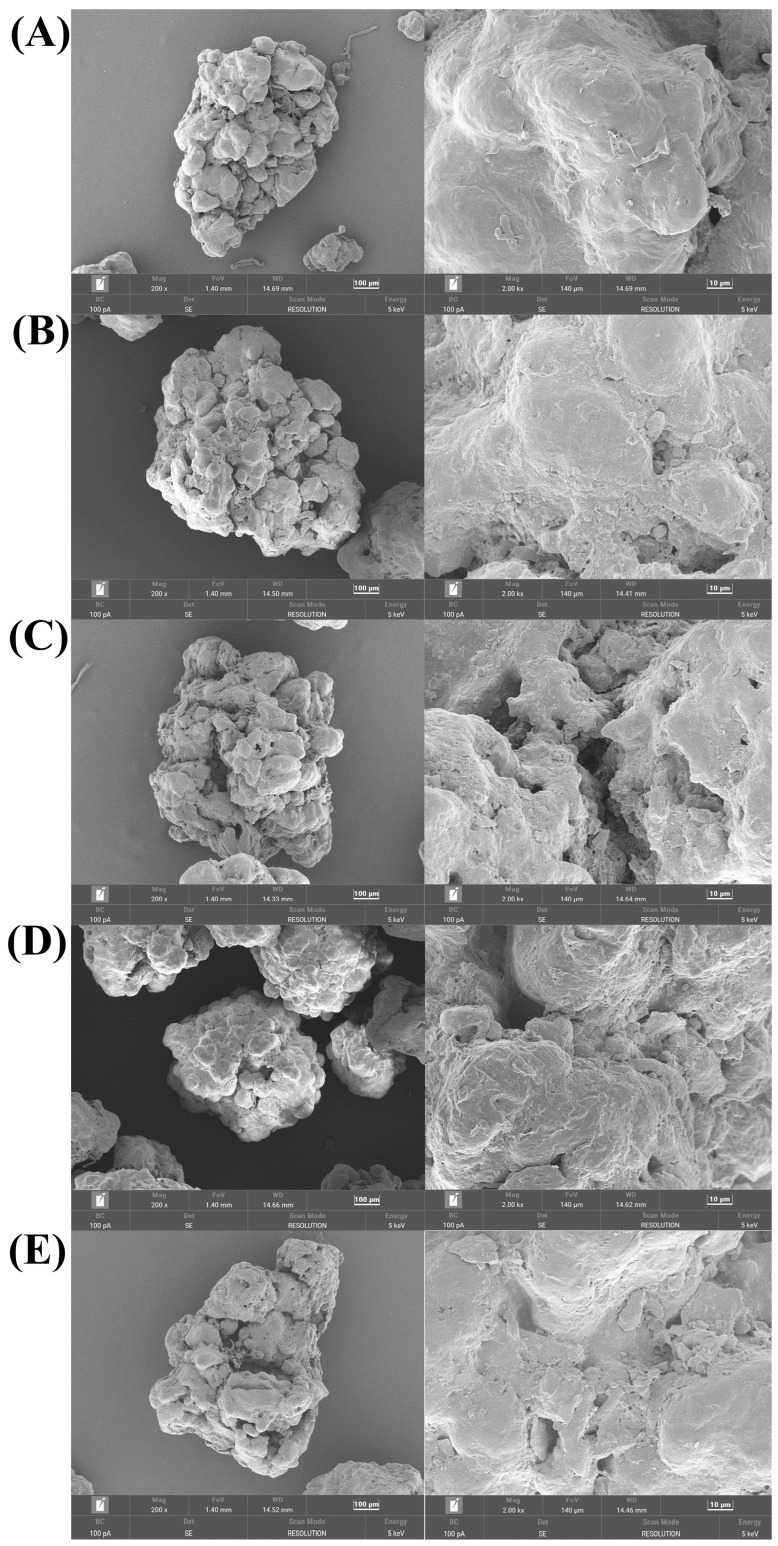
SEM images of untreated microplastics and microplastics in the culture of soils and soil– fertilizer systems. (**A**) PE; (**B**) A30PE-40; (**C**) B30PE-40; (**D**) A30PE-100; (**E**) B30PE-100.

Electron microscopy images showed some changes in the surface structure of microplastics after incubation in soil and fertilizer (Figure 6). The surface of the original microplastics was relatively flat, but after incubation, more wrinkles appeared on the surface of the microplastics. This is consistent with the results of Yang et al., where the microplastics in the manured soil had complicated weathered surfaces [14]. There was little difference in the morphologies of microplastics between soil incubation and soil and fertilizer incubation.

#### 3.3.2. Composition Variation

**Figure 7:**
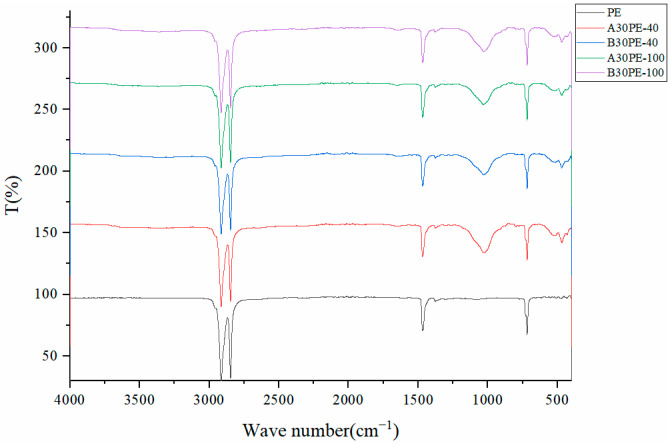
FTIR characteristics of untreated microplastics and microplastics in the culture of soils and soil–fertilizer systems.

FTIR spectroscopy was used to investigate the changes in the functional groups of microplastics before and after incubation, and the results are shown in Figure 7. All microplastic samples showed peaks at wavelengths of 2915, 2848, 1470, and 718 cm^−1^, which are characteristic peaks of PE plastics. The peaks at 2915 cm^−1^ and 2848 cm^−1^ correspond to the asymmetric and symmetric stretching vibrations of methylene, respectively. The peak at 1470 cm^−1^ corresponds to the bending vibration of methylene, and the peak at 718 cm^−1^ corresponds to the wobbling vibration of polymetallic methylene [15].

After incubation, additional peaks appeared at 1031 cm^−1^ in the microplastic samples, indicating changes in functional groups in PE after soil and soil and fertilizer incubation. The new peak near 1030 cm^−1^ may be attributed to residual polysaccharides on the biofilm [16], and some of the literature suggested that the peak can mainly be attributed to the stretching of C-O polysaccharide compounds [17].

## 4. Discussion

In this study, the addition of microplastics increased the relative abundance of humic acid-like components in soil DOM. The combined treatment with microplastics and organic fertilizer had a greater effect on the increase compared to the treatment with only microplastics, leading to a higher level of humification in the short term, which is consistent with the trend observed in the HIX (Figure 3 and Figure 4). During soil incubation, the addition of microplastics and organic fertilizer promoted the aggregation process of small-molecule substances in DOM. Studies have shown that the addition of microplastics and organic fertilizer can cause changes in soil DOM. On one hand, the changes in soil DOM composition and content may be related to the introduction of exogenous DOM by microplastics. Lee et al. observed that the main components of leachable DOM derived from microplastics were protein/phenol-like substances [18], suggesting that the changes in DOM in this study may also be partly due to the involvement of plastic-derived DOM. On the other hand, studies have also shown that the addition of organic fertilizer can cause the release of DOM into soil, playing a key role in the nutrient element release and carbon fixation in soil and downstream ecosystems [19,20].

Compared to the original soil, the addition of microplastics and the simultaneous addition of microplastics and fertilizer both increased the total carbon and soluble organic carbon content in the soil DOM. Liu et al. found that the addition of exogenous microplastics significantly increased the organic carbon content in soil, indicating a direct impact of microplastics on soil carbon elements [3], which is consistent with the results of this study. Exogenously added microplastics may interact with soil aggregates, affecting the structure and stability of aggregates, indirectly influencing soil physicochemical properties, and participating in the process of organic carbon cycling in soil. Moreover, as a high-carbon polymer, microplastics are also likely to biodegrade and directly participate in soil carbon cycling [21]. Additionally, organic fertilizer has a positive effect on increasing the content of water-soluble organic carbon in soil. Wang et al. found that the application of organic fertilizer can increase the content of soil humic substances, and promoted the conversion of protein components with simple molecular structures and low molecular weights in soil DOM into humic substances with more complex molecular structures and higher molecular weights, which could also be used by microorganisms [22].

Nitrogen is an important nutrient element that plays a crucial role in soil ecosystems. In this study, the addition of microplastics and organic fertilizer reduced the relative abundance of nitrogen in the soil, and this reduction was correlated with the size of the added microplastics. Qian et al. found that the total nitrogen content significantly decreased in farmland subject to long-term plastic film pollution compared to farmland without plastic film pollution [23]. Microplastics may affect the pore structure of soil through different particle sizes and alter the composition and structure of soil microbial communities, thereby influencing the metabolic cycle of nitrogen. The addition of microplastics to soil increased microbial community diversity and caused changes in microbial community composition. The addition of polyethylene microplastics inhibited the activity of leucine aminopeptidase in soil [24], while the addition of low-density polyethylene microplastics decreased the abundance of ammonia-oxidizing bacteria and nitrite reductase in soil [25]. It can also accelerate the diversity and succession of soil bacterial colonies [26], thus affecting the nitrogen cycling process in soil.

## 5. Conclusions

In this study, soil incubations of 40 days and 100 days were conducted to explore the effects of the addition of microplastics and organic fertilizer on the DOM composition and elemental components of soil. This study has demonstrated that the addition of microplastics increased the TOC concentration in soil DOM, and the increase was more pronounced in the treatment with the addition of organic fertilizer and microplastics. Microplastics increased the relative abundance of humic acid-like components in soil DOM, and the treatment with the simultaneous addition of microplastics and organic fertilizer promoted the humification level of the soil to a higher level in the short term.

## Figures and Tables

**Table 1:** The characteristics of the soil and fertilizer used in the incubation experiment.

Soil	TOC/mg·kg^−1^		C/%		H/%	N/%
	95.65		1.790		0.924	0.254
	Available nitrogen/mg·kg^−1^		Available phosphorus/mg·kg^−1^		Available potassium/mg·kg^−1^	pH
	72.772		95.314		276.346	7.41
	Organic matter/g·kg^−1^		<2 μm/%		2–50 μm/%	50–2000 μm/%
	19.773		4.91%		45.61%	49.48%
Fertilizer	C/%	H/%	N/%	Total nitrogen/mg·kg^−1^	Total phosphorus/mg·kg^−1^	Total potassium/mg·kg^−1^
	22.605	3.508	1.917	1.85	1.78	2.62

## Data Availability

The original contributions presented in the study are included in the article, further inquiries can be directed to the corresponding authors.
